# Physical activity prevents tumor metastasis through modulation of immune function

**DOI:** 10.3389/fphar.2022.1034129

**Published:** 2022-10-12

**Authors:** Aiping Zheng, Lei Zhang, Jiaqing Yang, Xiaomeng Yin, Tao Zhang, Xin Wu, Xuelei Ma

**Affiliations:** ^1^ Division of Biotherapy, Cancer Center, West China Hospital, Cancer Center, Sichuan University, Chengdu, China; ^2^ Head and Neck Oncology Ward, Cancer Center, West China Hospital, Cancer Center, Sichuan University, Chengdu, China; ^3^ Department of Obstetrics & Gynecology, Chengdu First People’s Hospital & Chengdu Integrated TCM & Western Medicine Hospital, Chengdu, China; ^4^ West China School of Medicine, West China Hospital, Sichuan University, Chengdu, China; ^5^ Head and Neck Oncology Ward, Division of Radiotherapy Oncology, Cancer Center, West China Hospital, Chengdu, China

**Keywords:** physical activity, tumor metastasis, microenvironment, immune function, immune cells

## Abstract

Metastasis is responsible for 90% of deaths in cancer patients. Most patients diagnosed with metastatic cancer will die within 5 years. PA is good for health and has become an emerging adjuvant therapy for cancer survivors. Regular moderate exercise substantially lowers the incidence and recurrence of several cancers, alleviates cancer-related adverse events, enhances the efficacy of anti-cancer treatments, and improves the quality of life of cancer patients. Revealing the mechanisms of PA inhibiting tumor metastasis could upgrade our understanding of cancer biology and help researchers explore new therapeutic strategies to improve survival in cancer patients. However, it remains poorly understood how physical activity prevents metastasis by modulating tumor behavior. The immune system is involved in each step of tumor metastasis. From invasion to colonization, immune cells interact with tumor cells to secret cytokines and proteases to remodel the tumor microenvironment. Substantial studies demonstrated the ability of physical activity to induce antitumor effects of immune cells. This provides the possibility that physical activity can modulate immune cells behavior to attenuate tumor metastasis. The purpose of this review is to discuss and summarize the critical link between immune function and exercise in metastasis prevention.

## 1 Introduction

Cancer metastasis is an important cause of death in cancer patients, with up to 90% of solid tumor patients dying from metastasis (Steeg, 2016). Most patients diagnosed with metastatic cancer will die within 5 years. The majority of current treatments concentrate on resection or elimination of primary tumor. Moreover, some clinical treatment strategies such as surgery have been demonstrated to aggravate cancer metastasis (Ma et al., 2019). Finding a safe and effective therapy for metastasis remains urgent.

Physical activity (PA) is good for improving physical and mental health. Nowadays, PA has become an important adjuvant therapy for cancer patients and has a remarkable influence on reinforcing conventional cancer therapies (Schmitz et al., 2019). Compared to other cancer treatments, PA has almost no toxic side effects, shows significant safety, and reduces treatment-related adverse events. According to the World Health Organization (WHO), cancer survivors should undertake at least 150–300 min of moderate intensity physical activity, or 75 min of vigorous intensity physical activity per week (Bull et al., 2020). Recently, PA has been shown to reduce the incidence of various cancers and improve the survival of cancer patients. A previous prospective cohort study reported that PA was negatively correlated with the incidence of post-menopausal breast cancer (Bellocco et al., 2016). Besides breast cancer, compelling evidence revealed that PA reduced the risk of additional cancer types, including colon, kidney, endometrial, bladder, esophageal and stomach cancers (Rock et al., 2020). Some prospective observational studies found that PA after cancer diagnosis may decrease cancer mortality, especially in colon (Meyerhardt et al., 2006), breast (Rock et al., 2020) and endometrial (Friedenreich et al., 2020) cancers. In addition, PA has been shown to improve the fatigue and quality of life (QoL) of cancer survivors, relieving anxiety and depression (Schmidt et al., 2015). However, whether PA has beneficial effects on metastasis is more attractive. Revealing the mechanisms of PA inhibiting tumor metastasis could upgrade our understanding of cancer biology and help researchers explore new therapeutic strategies to improve survival in cancer patients. In order to explore the potential mechanism linking PA with metastasis, some preclinical studies established various exercising animal models, especially running and swimming.

The immune system can effectively prevent the occurrence, development and metastasis of primary tumors through immune surveillance. Immune cells can recognize tumor-specific antigens and destroy cancer cells. Recently, some studies suggested that the modulation of the immune system through PA can significantly affect the exercise-dependent prevention of tumor metastasis (Lucia and Ramírez, 2016; Febbraio, 2017). Therefore, the aim of this review was to discuss and summarize recent findings that highlight the critical link between immune function and exercise in metastasis prevention.

## 2 Tumor metastasis and Physical activity

**FIGURE 1:**
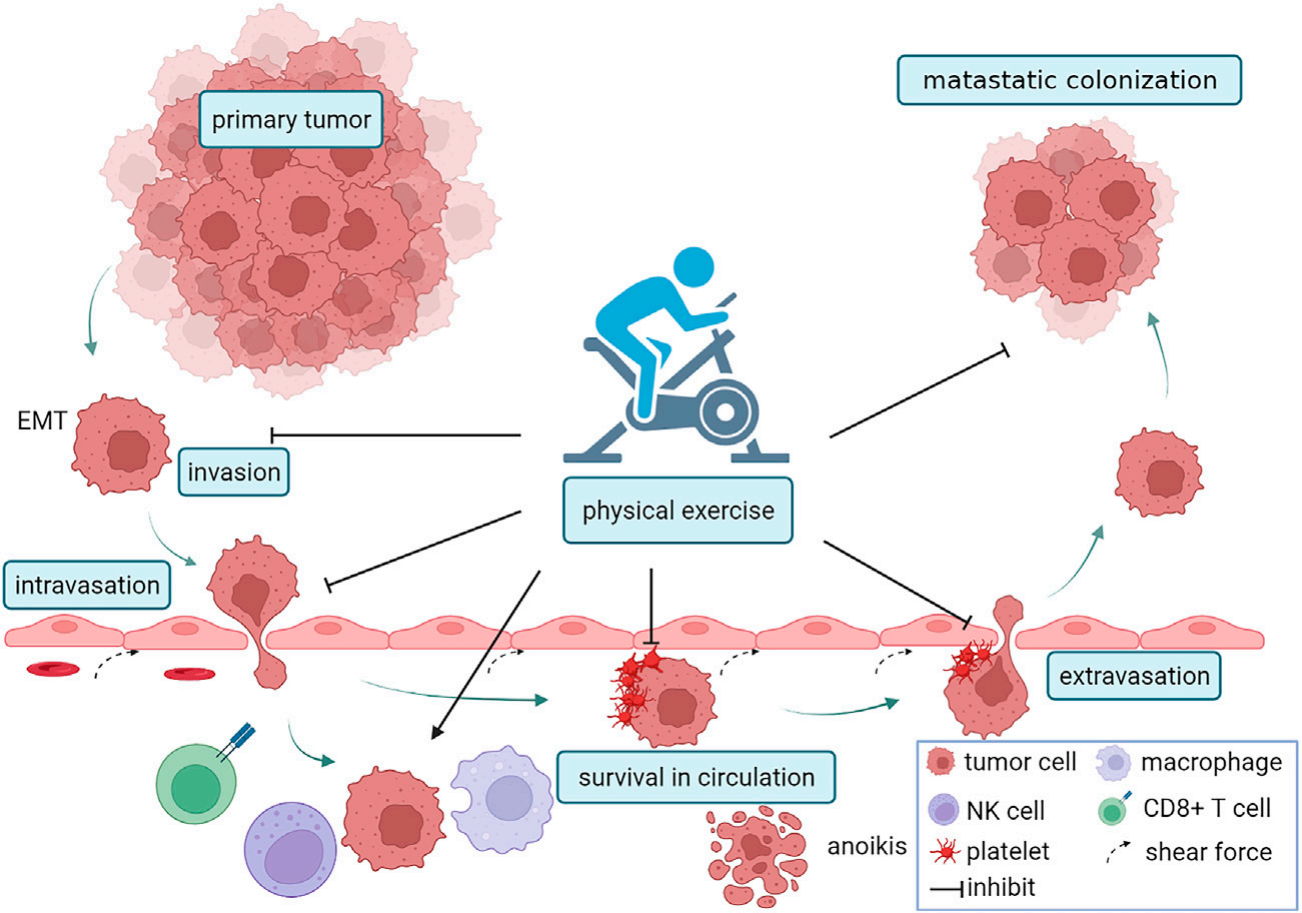
PA and the metastatic cascade. Firstly, PA reduced the invasion of tumor cells by inhibiting EMT. Next, PA reduced vascular permeability, inhibitd the interaction between endothelial cells and tumor cells, and suppressed intravasation. Exercise can also inhibit the survival of circulating tumor cells (CTCs) by increasing vascular shear force, recruiting macrophages, NK cells, and CD8+T cells, regulating metabolism and inducing anoikis. Exercise inhibitd platelet-tumor cells aggregates and the capacity of tumor cells to adhere to endothelial cells, which suppress extravasation. The figure was created with BioRender.com.

Tumor metastasis is a tangled and complicated process that can be categorized into five stages: invasion, intravasation, circulation, extravasation, and colonization. The cells were isolated from the primary tumor and acquired an invasive mesenchymal phenotype. In turn, invasive tumor cells infiltrate the blood vessels, a process closely related to vascular permeability and the interaction between tumor cells and endothelial cells. Once in circulation, invasive tumor cells are called circulating tumor cells (CTCs), and they confront challenges such as shear stress, oxidative stress, and immune surveillance. A few surviving CTCs invade blood vessels and colonize distant tissues, forming metastases. Emerging evidence suggests that physical exercise inhibits not only the invasion of tumor cells, but also the survival and distant colonization of circulating tumor cells. A schematic illustration of the association of exercise and metastasis is shown in Figure 1.

### 2.1 Physical activity and invasion

PA has been confirmed to attenuate the invasion of tumor cells *via* inhibiting epithelial-mesenchymal transition (EMT). A study showed that voluntary exercise led to an intratumor increase in E-cadherin levels and an intratumor decrease in the nuclear levels of *β*-catenin in ApcMin/+ mice (Ju et al., 2008). As is known to all, decreased expression of E-cadherin and increased expression of vimentin are the main characteristics of EMT. PA regulates multiple pathways to attenuate EMT. Moderate swimming could suppress EMT induced by TGF-β in transplanted hepatocellular carcinoma cells *via* promoting dopamine receptor 2 (DR2) activity (Zhang et al., 2016a). High-performance sports and resistance training can induce skeletal muscle to release myokine irisin. Irisin could inhibit EMT and invasion of tumor cells *via* the PI3K/Akt/Snail pathway has been demonstrated (Shao et al., 2017). Another study reported that irisin could be relevant to the activation of AMPK (Tang et al., 2016). Irisin downregulated the mTOR pathway and inhibited EMT of human pancreatic cancer cells *via* activating the AMPK (Liu et al., 2018). Moreover, irisin reversed the IL-6 induced EMT and downregulated the expression of MMP-2 by suppressing the STAT3/Snail signaling pathway (Kong et al., 2017).

### 2.2 Physical activity and intravasation

Physical exercise could influence intra-tumor angiogenesis by altering vascular epithelial growth factor (VEGF) in serum and tumor tissue. In prostate cancer, exercise induced the upregulation of HIF-1α and VEGF *via* activating MEK/MAPK and PI3K/mTOR signaling pathway, which is associated with a shift to tumor vascular normalization and inhibition of tumor metastasis (Jones et al., 2012). Data from ultrasonographic and thermographic also indicated higher vascularization of mammary tumors in exercised rats (Faustino-Rocha et al., 2017). The hypoxia and high permeability of the intratumoral vasculature also promote the intravasation of tumor cells. Physical exercise enhances tumor perfusion, diminishes hypoxia and transforms an aggressive tumor phenotype with abnormal leaky tumor vasculature to a weakly invasive tumor phenotype with normalized and mature vasculature (McCullough et al., 2014). Previous studies have demonstrated that physical exercise increases microvessel density and vessel maturity (Jones et al., 2012). In Ewing sarcoma, exercise modulated S1PR1 and S1PR2 expression, remodelling vasculature to reduce vessel hyperpermeability.

### 2.3 Physical activity and survival of circulating tumor cells

CTCs exposed to the circulation need to face various physical and biological stressors such as shear force, immune system surveillance, anoikis, and so on. Only a small portion (0.1%) of CTCs survive, and they have a relatively short half-life of about 1.0–2.4 h in circulation. Many studies have shown that exercise reduced CTCs in cancer patients. For instance, using a microfluidic antibody-mediated capture device to quantify CTCs inside venous blood of stage I-III colon cancer patients, researchers found that exercise led to a significant decrease in CTCs (Brown et al., 2018a). Exercise leads to an obvious increase in vascular shear force. During moderate-intensity exercise, the hemodynamic shear force can increase to 60 dyn/cm^2^ in human arteries and 5.2–6.2 dyn/cm^2^ in human veins (Tanaka et al., 2006). A previous study investigated the impact of hemodynamic shear force on the CTCs survival. The result revealed that high shear stress of 60 dyn/cm^2^ at intensive exercise killed more than 90% of CTCs within the first 4 h of circulation, contrasted with low shear stress of 15 dyn/cm^2^ at the resting state only killed 48% of CTCs (Regmi et al., 2017). Anoikis resistance played an important role in maintaining the survival of CTCs within circulation. HIF-1α protected CTCs from anoikis cell death by keeping an EMT state of CTCs (Majidpoor and Mortezaee, 2021). In untrained humans, acute exercise induced a transient increase of HIF-1α levels, while regular endurance exercise steadily reduced HIF-1α (Lundby et al., 2006; Wu et al., 2020a). Hippo signaling pathway has also been reported to be correlated with anoikis resistance. In metastatic breast cancer, up-regulated expression of zinc finger protein 367 (ZNF367) inhibited Hippo signaling pathway, giving rise to anoikis resistance and increased CTCs in circulation (Wu et al., 2020b). Exercise-conditioned sera could activate the Hippo signaling pathway and increase the inactivation of YAP (Baldelli et al., 2021). Exercise-induced epinephrine and norepinephrine also activated the tumor suppressor Hippo signaling pathway and promoted the phosphorylation of YAP. The phosphorylation then contributed to the sequestration of YAP in cytoplasm, which deterring the induction of tumor cell proliferation and survival by target genes (Dethlefsen et al., 2017). The effects of exercise on immune surveillance will be described in detail later.

### 2.4 Physical activity and extravasation

Surviving CTCs must arrest in the circulation and then start extravasation. In a previous study, long-term exercise led to a consistent lower retention of tumor cells in the pulmonary capillary bed compared with sedentary mice (MacNeil and Hoffman-Goetz, 1993a). Similarly, another study detected the radioactivity of ^51^Cr labelled CIRAS 1 tumor cells in lungs, liver, spleen and kidney. Researchers found that exercising mice showed a lower retention of radioactivity in secondary organs after tumor cells were injected into a tail vein (Hoffmann-Goetz et al., 1994).

### 2.5 Physical activity and colonization

Exercise may change the microenvironment of the major sites of metastases to inhibit the process of colonization. Recently, a study reported that exercise suppressed ovarian cancer colonization in the peritoneal cavity (Morrisson et al., 2021). The secretion of CCL2 and IL-15 had a significant increase in the peritoneal fluid of exercised mice. CCL2 can recruit macrophages and enhance their cytotoxicity. IL-15 can increase the reactivity of NK cells and CD8^+^Tcells in the peritoneal environment. Exercise also decreased the level of CCL22, VEGF, and CCL12 in peritoneal fluid. These cytokines lead to an immunosuppressive microenvironment by recruiting MDSC and Treg cells. Lung is a common metastasis site in malignant tumors, and it is also dramatically modulated by exercise. A further mechanism exploration found that exercise elevated antitumor cytotoxicity of alveolar macrophages by increasing the levels of tumor necrosis factor or reactive nitrogen intermediates (Davis et al., 1998). In order to successfully colonize the bone, tumor cells must escape the dormancy and keep proliferation. Tumor–osteoblast interactions have been proved to promote the dormancy of tumor cells. Physical exercise activated Cx43 hemichannels, and mechanically stimulated osteocytes to secrete Wnt and OPN (Fan et al., 2020), enhancing osteoblast activity and promoting the dormancy. Simultaneously, exercise increased the release of ATP from osteocytes (Genetos et al., 2007). The ATP-rich tumor microenvironment has been reported to suppress the proliferation of various tumor cells.

## 3 Tumor metastasis and immune function

The immune system can be divided into natural and adaptive immunity, and these two immune responses work synergistically to protect the organism (Wang et al., 2020). Natural immunity, also called innate immunity, is a semi-specific and extensive form of immunity. Natural immunity includes multiple immune cells and soluble factors and plays important roles in battling against pathogens. For example, neutrophils, macrophages, dendritic cells (DCs), natural killer (NK) cells, complement proteins and antimicrobial peptides (Janeway and Medzhitov, 2002). Adaptive immunity, also called acquired immunity or specific immunity, is a type of immune response that is generated by contact with a specific pathogen that can be recognized and initiated against the specific pathogen (Bonilla and Oettgen, 2010). Adaptive immunity consists mainly of T and B lymphocytes. T cells mediate cellular immune responses, while B cells are closely associated with the humoral immune response.

In the process of tumor metastasis, most cytotoxic innate and adaptive immune cells can synergistically control tumor behavior. A large number of cytotoxic immune cells such as NK and CD8^+^ T cells infiltrated around the primary tumor to eliminate many immunogenic cancer cells (Pagès et al., 2010). Natural killer (NK) cells can mediate tumor cells apoptosis *via* releasing granzyme B- and perforin. Cytotoxic CD8^+^ T cells kill tumor cells by secreting TNF-α and IFN-γ, while CD4^+^ T cells produce multiple cytokines to boost anti-tumor immune responses (Swann and Smyth, 2007; Ostrand-Rosenberg, 2008). The high levels of NK cells and cytotoxic T cells infiltration around the tumor are associated with better prognosis in cancer survivors (Nelson, 2008). CTCs are particularly sensitive to circulating immune cells. Circulating immune cells can directly and indirectly affect the viability of CTCs to control cancer metastasis (Dianat-Moghadam et al., 2021). The recruitment of cytotoxic M1 macrophages and N1 neutrophils, NK cells and mature DCs can all contribute to the elimination of CTCs.

However, some immunosuppressive cells such as myeloid-derived suppressor cells (MDSC) and regulatory T cells (Tregs), can secrete multiple cytokines and proteases to reshape the tumor microenvironment and promote immune escape, thereby promoting tumor metastasis (Smith and Kang, 2013). As tumors progress, cancer cells can secret multiple cytokines such as IL-4 and IL-13 to induce polarization of M2 macrophages and N2 neutrophils, which contributes to angiogenesis, extracellular matrix (ECM) remodeling and immune evasion. In addition, immature DCs also play important roles in facilitating tumor metastasis (Gonzalez et al., 2018).

## 4 Physical activity-dependent modulation of immune cells

**FIGURE 2:**
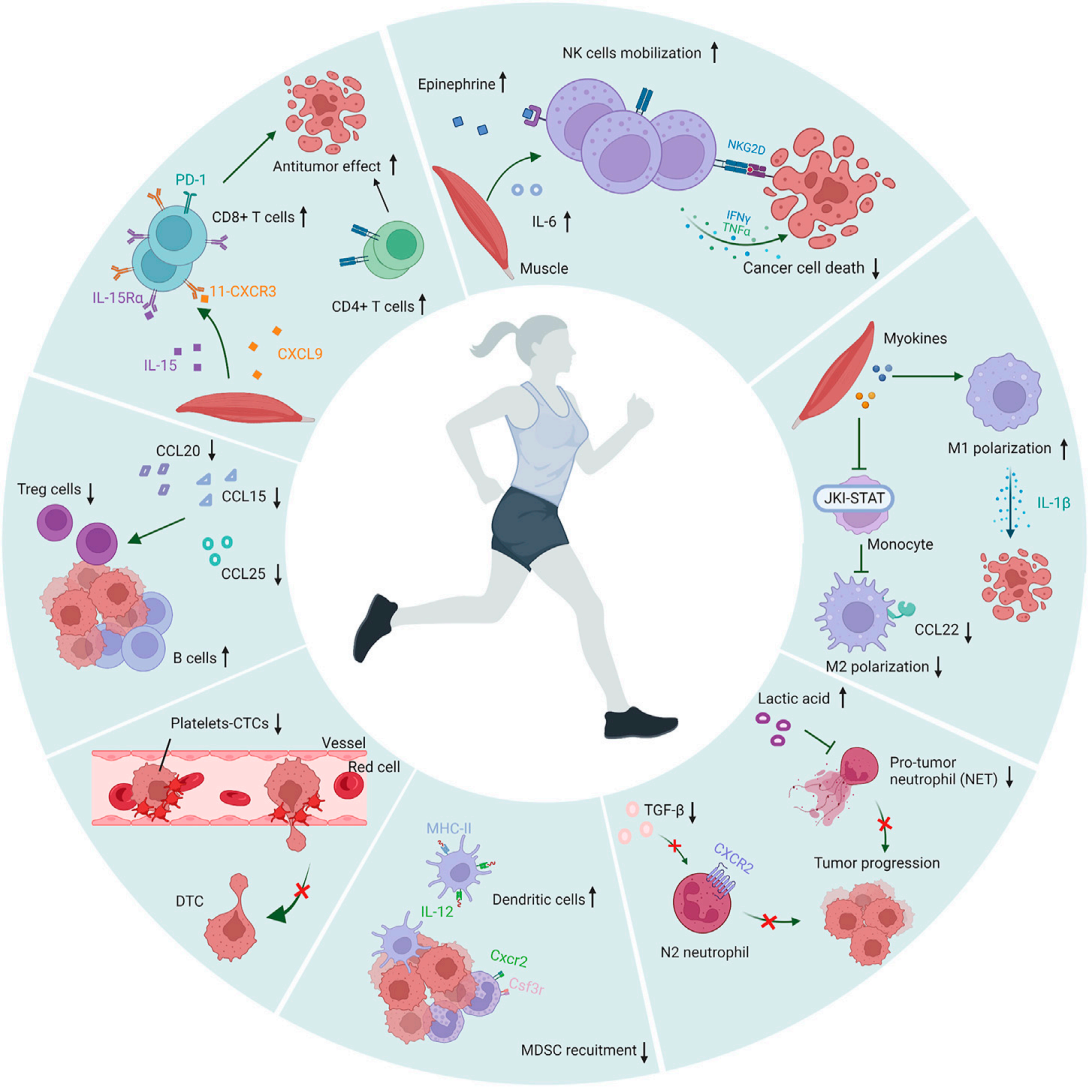
Modulation of immune cells during exercise. During physical activity, the numbers and antitumor effects of NK cells, dendritic cells, T cells and B cells were increased, the polarization of M2 macrophages and N2 neutrophils are inhibited, and the recruitment of MDSC and Treg was suppressed. Moreover, PA inhibited the formation of platelet-CTCs aggregates and reduced the adhesion of platelets to endothelial cells. The figure was created with BioRender.com.

PA has a positive effect on the human immune system, especially the innate immune system. During PA, cytotoxic immune cells are mobilized into the circulation through stress-induced shear stress and adrenergic signaling (Idorn and Hojman, 2016). This mobilization is not to induce the body to produce a new generation of immune cells, but to recruit the existing storage of immune cells (Walsh et al., 2011). According to numerous studies, chronic and acute physical exercise show significant responses in terms of immune cells redistribution, activity and function in cancer patients. The intensity and duration of exercise also affects the redistribution of immune cells to the circulation (Robson et al., 1999; Freidenreich and Volek, 2012; Bigley and Simpson, 2015). In some tumor-bearing animal models, exercise led to an increase in the number and function of effector cells and a decrease in immunosuppressive cells (Thompson et al., 2010; Hagar et al., 2019). Here we discuss the effect of exercise on multiple immune cells in the process of metastasis. A schematic illustration of the association of exercise and immune cells is shown in Figure 2.

### 4.1 Physical activity and natural immune cells

As the first-line defenders against pathogens, natural immune cells are hot topics to exercise immunology.

#### 4.1.1 Natural killer cells

Among natural immune cells, NK cells are the most responsive to exercise, showing exercise-dependent acute mobilization. The number of NK cells can be increased to more than six-fold during a brief stair climb, without immediate functional decrease after rest (Millard et al., 2013). This rapid mobilization of NK cells is mainly related to the exercise intensity-dependent changes in catecholamine concentrations (Kappel et al., 1991). NK cells have the most abundant *β*-adrenergic receptors in all immune cells (Landmann, 1992). Systemic administration of epinephrine mimics the exercise-induced increase in circulating NK cell infiltration, while nonselective and selective β1-and β2-blockers can block this mobilization effect from exercise (Murray et al., 1992). During acute PA, muscle-derived cytokines such as IL-15, IL-7, and IL-6 are also involved in NK cells activation (Benatti and Pedersen, 2015). However, after long-term exercise, the number of circulating NK cells was reduced, which may due to tissue migration or re-marginalization (Timmons and Cieslak, 2008). PA not only increases the number of circulating NK cells, but also enhances their antitumor activity. A previous study has demonstrated that exercise enhanced splenic NK cells activity in tumor-bearing mice (MacNeil and Hoffman-Goetz, 1993b). Another study has found that mice randomly assigned to the voluntary wheel had an obvious increase in NK cells infiltration in various tumor models (melanoma, Lewis lung cancer and liver cancer), leading to reductions in tumor incidence, growth and metastases. Exercise recruits NK cells *via β*-adrenergic signaling and induces muscle-derived IL-6 to redistribute and activate NK cells. Moreover, the expression levels of NK cell-related activating receptor ligands (NKG2D, MULT1, H60a, and Clr-b) also had an increase in the tumors of running mice, revealing that exercise worked on the mobilization of NK cells and the formation of NK cell activated tumor microenvironment (Pedersen et al., 2016).

#### 4.1.2 Macrophages

Macrophages also play a pivotal role in controlling tumor metastasis. M1 macrophages have the capacity to diminish a large number of CTCs, while M2 macrophages are related to the promotion of tumor metastasis. Exercise can enhance antitumor macrophage cytotoxicity and suppress the polarization of macrophages to the M2 (Davis et al., 1998; Goh et al., 2012). Short-term moderate-exercise training led to an increase in macrophages antitumor cytotoxicity and decreased the lung tumor metastases of injected B16 melanoma cells (Murphy et al., 2004). Another recent study using a triple-negative breast cancer mouse model reported that exercise reduced M2 macrophage polarization by inhibiting the JAK-STAT signaling pathway, thus decreasing lung cancer metastasis (Kim et al., 2020). M2 macrophages secreted chemokine CCL22, which attracted CCR4-expressing Tregs in circulation toward the CCL22 gradient, thus facilitating the recruitment of Tregs. Exercise contributed to a significant decrease in CCL22 mRNA expression in M2 macrophages and resulted in a reduction in Treg recruitment, which delayed invasive breast cancer progression and metastasis in polyoma middle T oncoprotein (PyMT) transgenic mouse (Goh et al., 2013). Similarly, in an ApcMin/+ mouse model, the mRNA expression of M2 related macrophage markers such as CD206, CCL22 and Arg consistently decreased in exercise mice (McClellan et al., 2014).

#### 4.1.3 Neutrophils

Some recent studies revealed that neutrophils promoted the metastasis potential of cancer cells. In circulation, neutrophils induced the aggregation of tumor cells to improve the survival rate of CTCs (Szczerba et al., 2019). Neutrophil extracellular traps (NETs) released by neutrophils was also demonstrated to enhance the tumor metastasis. Some studies have shown that exercise can inhibit NETs formation (Shi et al., 2020). The accumulation of exercise-induced lactic acid decreases the release of NETs in serum (Shi et al., 2019). A recent study reported that exercise mitigated liver ischemia-reperfusion injury derived inflammatory responses and metastasis *via* inhibiting neutrophil recruitment and diminishing NETs release in the mouse model of colorectal adenocarcinoma (Yazdani et al., 2021). Nevertheless, tumor-associated neutrophils (TANs) had a two-sided effect in the progression of tumor (Uribe-Querol and Rosales, 2015). Some previous reports demonstrated that neutrophils directly destroyed tumor cells both *in vitro* and *in vivo* (Uribe-Querol and Rosales, 2015). In metastatic breast cancer and renal carcinoma, the tumor cells produced CCL2 and IL-8 that induced neutrophil recruitment to inhibit lung metastasis, respectively (Granot et al., 2011; López-Lago et al., 2013).

Similar to the M1 and M2 phenotypes of macrophages, neutrophils also have N1 and N2 polarization states. N1 neutrophils have anti-tumor function by secreting type I interferon and inducing NK cells to secret IL-18. N2 neutrophils secrete multiple molecules such as arginase and peroxidase to inhibit T cells and NK cells functions, which promote tumor metastasis. TGF-β derived from tumor microenvironment can induce the activation of N2 neutrophils (Fridlender et al., 2009). PA has been demonstrated to inhibit the expression of TGF-β in tumor tissue (da Silva Alves et al., 2020), which attenuates the polarization of N2 neutrophils.

#### 4.1.4 Dendritic cells

DCs play a key role in eliminating and controlling tumor progression. In human exercise studies, PA can increase the number of DCs in the peripheral blood circulation (Ho et al., 2001; LaVoy et al., 2015). Further study showed that exercise upregulated the expression of MHC II and IL-12 on DCs in animal models (Liao et al., 2006; Chiang et al., 2007). A previous study investigated the composition of DCs subpopulations mobilized in response to acute aerobic exercise. The findings showed that exercise preferentially mobilized plasmacytoid DCs into peripheral blood to enhance immune surveillance (Brown et al., 2018b). However, there are few studies investigating the effects of exercise on DCs in cancer patients, and more research is needed in the future.

#### 4.1.5 Myeloid-derived suppressor cells

MDSCs are effective inhibitory factors of immune function and contribute to the immune escape. Augmented ROS produced by MDSCs induced the upregulation of Notch1 in CTCs through the ROS-NRF2-ARE axis, thus enhancing CTCs metastatic traits (Sprouse et al., 2019). Recently, a preclinical study found that PA reduced the tumor-induced accumulation of MDSCs and delayed the tumor growth in a mouse model of triple negative breast cancer (Wennerberg et al., 2020). In 4T1 tumor-bearing mice, voluntary wheel running potently relayed the accumulation of IMCs/MDSCs in the spleen, blood, and tumor. Moreover, these effects led to a reduction in the number of metastatic lung nodules in exercising mice (Garritson et al., 2020). Another previous study also showed that the combination of PA and energy restriction decreased MDSC accumulation by restraining myelopoiesis and/or the mobilization and transportation of MDSCs to secondary sites (Turbitt et al., 2019). In a mouse model of pancreatic cancer, PA diminished MDSC *via* downregulating the expression levels of Cxcr2 and Csf3r on myeloid cells (Kurz et al., 2022). These findings suggested that PA was beneficial to inhibiting tumor progression and metastases *via* suppressing MDSCs accumulation.

#### 4.1.6 Platelets

Platelets activation plays an essential role in elevating the survival rate of the CTCs. Activated platelets adhere to CTCs to protect the tumor cells from various stressors in circulation. Moreover, aggregates of platelets and CTCs have been shown to inhibit NK cells antitumor cytotoxicity in vitro-model. Some reports have found that PA affected the clearance of CTCs by modulating platelets activity. Compared with sedentary mice with breast cancer, exercising mice had a lower number of circulating platelets (Smeda et al., 2017). In patients with nasopharyngeal carcinoma, moderate-intensity exercise decreased the formation of platelets-CTCs aggregates and minimized the risk of metastasis (Wang et al., 2007). Another study by the same team found that warm-up exercise before severe exercise reduced platelet-impeded cytotoxicity of NK cells to nasopharyngeal carcinoma cells (Wang et al., 2009).

The activation of platelets is also critical for CTCs to extravasate. Adhesion molecules on activated platelets can gather CTCs to securely adhere to the activated vascular endothelial cells. PA might be related with the downregulation of adhesion molecules on platelets and endothelial cells (ECs), such as P-selectin and epithelial cell adhesion molecule-1 (EPCAM-1) (Wang et al., 2005; Souza et al., 2017). In P-selectin-deficient mice, lung metastasis was significantly reduced post injection of tumor cells (Borsig, 2004). Nevertheless, the positive effects of exercise might be limited by intensity. Some investigations have found that strenuous exercise promoted platelets aggregation and the capacity of CTCs to adhere to ECs for sedentary healthy humans (Chen et al., 2009), yet moderate exercise inhibited platelets aggregation and adhesiveness (Wang and Liao, 2004; Wang et al., 2005; Wang, 2006).

### 4.2 Physical activity and adaptive immune cells

Adaptive immune cells consist mainly of T and B lymphocytes. In general, exercise-induced lymphocytosis is proportional to the duration and intensity of exercise.

#### 4.2.1 Cytotoxic T cells

Cytotoxic T cells recognize and diminish CTCs by specifically identifying mutation-induced neoantigens. A study using breast cancer mice found that acute exercise caused a transient increase in CD8^+^ T cells. Exercise-induced decrease in tumor growth was contingent on the levels of CD8^+^ T cell in circulation. And key metabolites that muscles released into the blood during exercise, including lactate, made CD8^+^ T cells more effective. Moreover, these super-effective CD8^+^ T cells extracted from exercising mice showed better antitumor efficacy when transferred to sedentary mice (Rundqvist et al., 2020). Recently, a preclinical study also suggested that PA can increase the infiltration and effector function of CD8 T cells in breast tumors. Further investigation showed that CXCL9/11-CXCR3 pathway is required for the CD8^+^ T cell-mediated antitumor effect of PA (Gomes-Santos et al., 2021). In a mouse model of pancreatic cancer, PA activated IL-15/ IL15Rα pathway to promotes activation of CD8^+^ T cells (Kurz et al., 2022). Notable, exercise-induced IL-15Rα CD8^+^ T cells selectively upregulate checkpoint PD-1, which contributes to increase sensitivity to chemotherapy.

CD4^+^ T cells also play a central role in antitumor immune response. Similar to CD8^+^ T cells, a temporary increase in CD4^+^ T cells was detected after resistance exercise (Natale et al., 2003). In a mouse model of hepatocellular carcinoma, exercise enhanced immunity by raising CD4^+^ T lymphocytes in peripheral blood (Zhang et al., 2016b).

#### 4.2.2 Tregs

Tregs effectually inhibit the activation and proliferation of CD8^+^ T cells, which are considered to be the important barriers to impede the effect of anti-tumor immunity. An increased number of Tregs indicated a higher CTCs-positive rate and contributed to a poorer clinical outcome in cancer patients (Xue et al., 2018). A previous study demonstrated that endurance exercise suppressed the recruitment of Tregs and relayed the tumor growth in breast cancer. Exercise led to a greater tumor immune response by increasing the ratio of CD8/Tregs (Hagar et al., 2019). PA induced the downregulation of chemokines such as CCL5, CCL20 and CCL25, which were closely associated with the recruitment of Tregs (Turbitt et al., 2019).

#### 4.2.3 B cells

The role of B cells in cancer progression is much less understood than that of T cells. Growing evidence suggested that tumor-infiltrating B cells may exert both tumor suppressive and tumor promoting effects (Gu et al., 2019; Xu et al., 2022). During exercise, circulating B-cell counts increased mildly immediately and in proportion to exercise duration and intensity (Ronsen et al., 2001). However, there are few studies elucidating the effects of exercise on B cells immune function in cancer patients.

### 4.3 Additional exercise effects

Physical exercise improves blood perfusion and hypoxia, which also affect immune function. Hypoxia induces overexpression of connexin 43 in tumor cells, leading to degradation of NK cell immune synapses and impairing NK cell killing activity (Tittarelli et al., 2015). Improving intra-tumor hypoxia can indirectly increase the cytotoxicity of tumor-infiltrating NK cells. Moreover, PA promotes normalization of intratumoral vessels and blood perfusion, which can increase the accessibility of immune cells and delivery of antitumor drugs.

## 5 Deficiencies and prospects

Existing preclinical and clinical studies have demonstrated that PA, particularly regular moderate exercise, plays a beneficial role in tumor metastasis. The immune system is highly responsive to exercise, which may lead to beneficial effects on tumor metastasis. During exercise, a large number of cytotoxic immune cells with antitumor functions are mobilized into circulation to kill CTCs. To be sure, the mechanisms of exercise modulating immune cells are extensive and diverse. However, the exploration of the potential mechanisms underlying the beneficial effect of exercise on immune cells is still in its early stages. The review analyzed that PA can control metastasis by regulating immune function. As the understanding of the mechanisms by which PA effects tumor metastasis continues to improve, new therapeutic strategies will be identified and validated, potentially contributing to improve survival in cancer patients.
